# The effect of mislabeled phenotypic status on the identification of mutation-carriers from SNP genotypes in dairy cattle

**DOI:** 10.1186/s13104-017-2540-x

**Published:** 2017-06-26

**Authors:** Stefano Biffani, Hubert Pausch, Hermann Schwarzenbacher, Filippo Biscarini

**Affiliations:** 1 IBBA-CNR, Via Einstein-Loc. Cascina Codazza, 26900 Lodi, Italy; 2 Technische Universität München, Liesel-Beckmann Straße 1, 85354 Freising-Weihenstephan, Germany; 3 ZuchtData EDV Dienstleistungen GmbH, Dresdner Straße 89/19, 1200 Wien, Austria; 4 Division of Infection & Immunity, School of Medicine, Cardiff University, Heath Park, CF14 4XN Cardiff, UK; 5 AIA: Associazione Italiana Allevatori, Via Giuseppe Tomassetti 9, 00161 Rome, Italy

**Keywords:** Noisy data, Genomic predictions, SNP genotypes, *Bos taurus*, Animal genomics, Classification, Phenotyping errors

## Abstract

**Background:**

Statistical and machine learning applications are increasingly popular in animal breeding and genetics, especially to compute genomic predictions for phenotypes of interest. Noise (errors) in the data may have a negative impact on the accuracy of predictions. The effects of noisy data have been investigated in genome-wide association studies for case–control experiments, and in genomic predictions for binary traits in plants. No studies have been published yet on the impact of noisy data in animal genomics. In this work, the susceptibility to noise of five classification models (Lasso-penalised logistic regression—Lasso, K-nearest neighbours—KNN, random forest—RF, support vector machines with linear—SVML—or radial—SVMR—kernel) was tested. As illustration, the identification of carriers of a recessive mutation in cattle (*Bos taurus*) was used. A population of 3116 Fleckvieh animals with SNP genotypes on the same chromosome as the mutation locus (BTA 19) was available. The carrier status (0/1 phenotype) was randomly sampled to generate noise. Increasing proportions of noise—up to 20%— were introduced in the data.

**Results:**

SVMR and Lasso were relatively more robust to noise in the data, with total accuracy still above 0.975 and TPR (true positive rate; accuracy in the minority class) in the range 0.5–0.80 also with 17.5–20% mislabeled observations. The performance of SVML and RF decreased monotonically with increasing noise in the data, while KNN constantly failed to identify mutation carriers (observations in the minority class). The computation time increased with noise in the data, especially for the two support vector machines classifiers.

**Conclusions:**

This work was the first to assess the impact of phenotyping errors on the accuracy of genomic predictions in animal genetics. The choice of the classification method can influence results in terms of higher or lower susceptibility to noise. In the presented problem, SVM with radial kernel performed relatively well even when the proportion of errors in the data reached 12.5%. Lasso was the second best method, while SVML, RF and KNN were very sensitive to noise. Taking into account both accuracy and computation time, Lasso provided the best combination.

**Electronic supplementary material:**

The online version of this article (doi:10.1186/s13104-017-2540-x) contains supplementary material, which is available to authorized users.

## Background

**Fig. 1:**
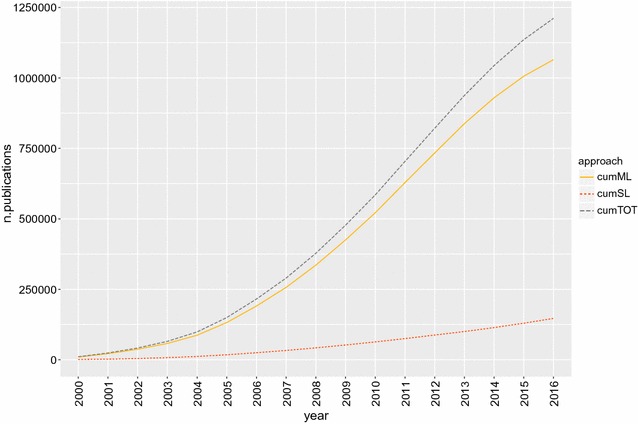
Cumulative number of machine and statistical learning-related publications over time. From Google Scholar queries for “machine learning” and “statistical learning” publications between 2000 and 2016 [machine learning (ML)* solid gold line*, statistical learning (SL)* dotted red line*, total* dashed dark gray line*]

In data science, statistical and machine learning approaches are used to identify patterns within data, with the primary objective of making predictions on future or unobserved data. Their popularity has increased with the size of available data: the advent of “big data” [1] has outdated many classical data analysis and statistical approaches. From a search on Google Scholar the number of publications related to statistical and machine learning increased from 10,690 in year 2000 to 1,211,400 in year 2016, with a peak rate between years 2011 and 2013, to then continue to increase at a slower pace (Fig. 1). Statistical and machine learning are nowadays applied to many different areas like Web Search, spam filters, recommender systems, ad placement, credit scoring, fraud detection [2–5], and to diverse biological disciplines, like drug development, DNA sequence analysis, cell biology and animal genetics [6–9].

A common learning task is classification (binomial or multinomial), where the objective is to build a classifier that can correctly predict the class of a new object given some training examples of known objects [10].

Machine learning methods may be susceptible to biases, especially if we consider that the training data can contain errors. Errors in the data are known as noise, and can arise because of different reasons (e.g. instrument errors, quantization errors, environmental noise, model mis-specification, human errors, inherent randomness in the physical processes): the consequence is that the classifier learns from a distorted version of the actual data and its predictive ability will be biased upwards or downwards, or randomly unreliable [11, 12].

In the field of genomics, genotypes are typically used together with phenotypes to either detect associations or make whole-genome predictions [13, 14]. Errors may be found in the genotypic and/or in the phenotypic data. The consequences of genotyping errors [15, 16], and of errors in the imputation of missing genotypes [17–20] on genome-wide association studies and genomic predictions have been addressed. Scientific literature on phenotypic errors in genomics is much scarcer. The effect of phenotype misclassifications on the statistical power of genome-wide association studies (GWAS) has been addressed in case–control studies in human medicine [21]. More recently, the influence of noisy data on the accuracy of whole-genome predictions has been examined in sugar beets [22]. No studies have been published yet on the impact of noisy phenotypes on genome-enabled predictions in human or animal genomics.

In this paper, the impact of randomly mislabeled observations on the accuracy of genomic predictions for binary traits is investigated. A cattle (*Bos taurus*) population with known carrier/non-carrier status for a harmful recessive genetic mutation was used for illustration. SNP genotypes were used to classify animals. Starting from a dataset with known mutation carrier status (no errors), increasing proportions of noisy labels were randomly generated, and the performance of different classification methods was measured.

## Methods

### Experimental data

SNP genotypes and mutation carrier status were available for a dairy cattle population of 3116 Fleckvieh animals. The mutation used for illustration is the *TUBD1* recessive mutation [23, 24] at the beginning of BTA19 (*Bos taurus* autosome 19). The TUBD1 mutation (and the associated BH2 haplotype: [25]) were reported to be associated with stillbirth and calf survival rate [26]. Animals were labeled as carriers or not of the mutation (coded as 1 or 0). There were 126 carriers (4.04%) and 2990 non-carriers (95.96%). All animals were genotyped with the Bovine SNP50 v2 (54K) Illumina BeadChip. Only the 1512 SNPs on BTA19 were used for the analysis. No individual animal had a call-rate lower than 95%. SNPs with a call-rate lower than 95% (195 SNP) were removed from the analysis. Residual missing SNP genotypes were imputed based on linkage disequilibrium, using the localized haplotype clustering imputation method implemented in the computer package “Beagle” v.3 [27]. Data for the present study were provided by ZuchData EDV-Dienstleistungen GmbH (Austria).

### Classification models

Five machine learning (ML) algorithms were used to identify mutation carriers from SNP genotypes: Lasso-penalised logistic regression (Lasso), Support Vector Machines using either a linear (SVML) or a radial (SVMR) kernel, K-nearest neighbours (KNN) and random forest (RF). In order to explore the effect of noisy labels on genomic classifications, 10 different scenarios were simulated. In each scenario an increasing proportion of noise was introduced by flipping independently the original carrier state. The following noise proportions were tested: 0% (original data with no errors), 1, 2.5, 5, 7.5, 10, 12.5, 15, 17.5 and 20%. For each proportion of mislabelled observations, the five classification models were tested.

#### Lasso-penalised logistic regression (Lasso)

The probability of carrying the mutation ($P(Y = 1|X) = p(x)$) was modeled as a linear combination of SNP genotypes in a logistic regression model:

1 $$\begin{aligned} logit(p(x_i))=\mu +\sum _{j=1}^m z_{ij}SNP_j \end{aligned}$$

where *p*(*x*) is the $P(Y = 1|X)$ for individual *i* with vector of SNP genotypes $x_i$; S$NP_j$ is the effect of the $j$th marker; $z_{ij}$ is the genotype of individual *i* at locus *j* (0, 1 or 2 for AA, AB and BB genotypes). The model in Eq. 1 was fitted by maximizing the corresponding Lasso-penalized log likelihood function [28]. The tuning parameter $\lambda$ controls the degree of regularization, and was specified through cross-validation. Logistic regression returns the log-odds of *p*(*x*) which are back-transformed to $P(Y = 1|X)$ through the cumulative distribution function of the logistic distribution (i.e. the logistic function). Individuals with $p(x) > / < 0.5$ were classified as carriers or not of the mutation.

#### Support vector machines (SVM)

 Two support vect//.oor machines (SVM) models were fitted for the classification of carriers and non-carriers of the mutation: with linear (SVML) and radial (SVMR) kernel functions. SVM maps the vector of SNP genotypes $x \in \mathcal {R}$ into a higher dimensional feature space $\phi (x) \in \mathcal {H}$ and constructs a decision boundary which is linear in $\mathcal {H}$, and possibly non-linear in $\mathcal {H}$. Animals are then classified into carriers and non-carriers of the mutation based on the width of the margin $\mathcal {M}$ and the sign of the classifier:

2 $$\begin{aligned} f(x)=\beta _0 + \sum _{i=1}^n \alpha _i K(x,x_i) \end{aligned}$$

The kernel function *K* has the form $K(x_i,x_{i'})=\sum _{j=1}^m x_{ij}x_{i'j}$ in SVML and $K(x_i,x_{i'})=exp \left( -\gamma \sum _{j=1}^m (x_{ij}-x_{i'j})^2 \right)$ in SVMR. The hyperparameters *C* (which controls the width of the margin $\mathcal {M}$) and $\gamma$ (which controls the degree of non-linearity in SVMR) were chosen so to minimize the classification error through cross-validation in the training set. A full description of SVM can be found in [29].

#### K-nearest neighbours (KNN)

 The predicted carrier/non-carrier status for animal $x_0$ was obtained by majority vote among the *K* closest neighbours. The neighbourhood was determined by Euclidean distances based on SNP genotypes, for each neighbour *i* over *m* SNP dimensions:

3 $$\begin{aligned} D_E=d(x_0,x_i)=\sqrt{\sum _{j=1}^m (x_{0j}-x_{ij})^2} \end{aligned}$$

The size of the neighbourhood K was determined through cross-validation in the training data.

#### Random forest (RF) classifier

 A large number of classification trees was built on $B=500$ bootstrapped samples of the data. Classification trees were decorrelated by using, at each node, a random subset *s* of the 1512 SNPs on BTA19. The size of the random feature subset *s* was optimized around $\sqrt{1512} \approx 39$ SNPs The final classifier was obtained by majority vote over the *B* classification trees:

4 $$\begin{aligned} \hat{f}_{avg}(x_i)=\frac{1}{B}\sum _{b=1}^B I\left(\hat{f}_b(x_i)=[0/1]\right) \end{aligned}$$

where $x_i$ is the vector of SNP genotypes for animal *i*, and $\hat{f}_b(x_i)$ is the prediction (carrier/non-carrier) from the classification tree built on the $b$th bootstrapped data sample. More details on random forest can be found in [30].

### Prediction accuracy

In order to compare the predictive ability of the five classifiers, the data were initially split in a training and a testing data set: $70\%$ of the observations used for training, $30\%$ of the observations used for testing. The training dataset (which contained increasing proportions of random noise) was used to tune the hyperparameters ($\lambda$ for Lasso; *C* and $\gamma$ for SVML and SVMR; *K* for KNN; *s* for RF) and train the classifier through a 10-fold cross-validation procedure: the hyperparameters that gave the lowest average balanced accuracy in the validation sets (the 10th fold, in turn) were selected. The final model was then applied to the testing set to predict the original carrier-non carrier status and measure the accuracy of classification. Prior to fitting the model, monomorphic and collinear (correlation >0.99) SNPs were edited out of the training set, to remove non-informative and redundant predictors and avoid problems due to linear dependencies. This procedure was repeated 10 times per each proportion of noise (0–20%), using different training and testing subsets each time. The following measures of classification accuracy were calculated in the testing data set: (1) accuracy (ACC): the proportion of the total number of correct predictions over the total test sample size; (2) true positive rate (TPR, sensitivity): the proportion of mutation carriers (positives) that were correctly identified, over the total number of carriers in the test set; and (3) true negative rate (TNR, specificity), the proportion of non-carriers (negatives) that were correctly identified over the total number of non-carriers in the test set. Results were averaged over replicates by noise proportion.

### Software

Data preparation and editing, and all statistical analysis were performed using the *R* programming environment v.3.2.3 [31], except missing genotype imputation, which was carried out with the computer package “Beagle” v.3.3.2 [27]. The R packages *glmnet* [32], *e1071* [33], *class* and *caret* [34] were used to fit the Lasso logistic regression, SVM with linear and radial kernels, KNN and RF classification models. The analyses were run on the bioinformatics platform at *PTP Science Park* (http://www.ptp.it), which includes a high performance computing cluster with 600 CPUs, 2.5 TB of RAM and 100 TB of storage space for archiving and back-up.

## Results

**Fig. 2:**
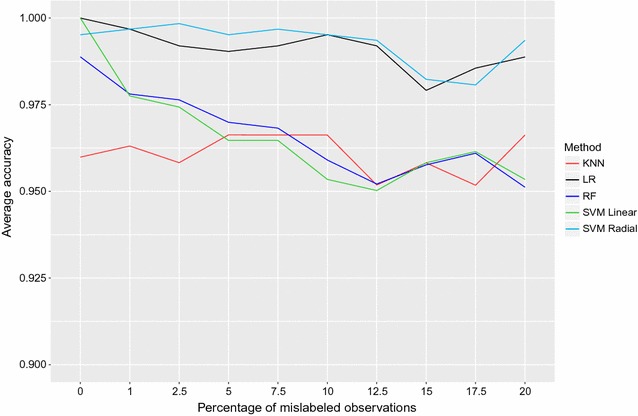
Total prediction accuracy (ACC) as a function of noise in the data. Proportion of observations (both carriers and non-carriers of the mutation) that were correctly identified by the five classification methods over the 10 proportions of errors introduced. Lasso* black*, SVML* green*, SVMR* light blue*, RF* blue*, KNN* red*

The total prediction accuracy for the five classification methods over the ten proportions of random errors introduced in the data is shown in Fig. 2. Total accuracy (ACC) was above 95% for all methods and proportions of errors. Lasso and SVML reached 100% accuracy with no errors in the data. When errors began to be introduced, the accuracy of SVML decreased, down to 95.02% with 12.5% errors. For Lasso and SVMR, ACC was above 99% from 0 to 12.5% errors in the data, dropping to 98% for 15 and 17.5% errors, and eventually relapsing back above 99% with 20% errors in the training set. With RF, ACC was 98.9% with no errors in the data and went down to 96.1% with 20% mislabelings. KNN gave a lower average ACC (95–96%) which remained fairly constant over different percentages of noise in the data.

**Fig. 3:**
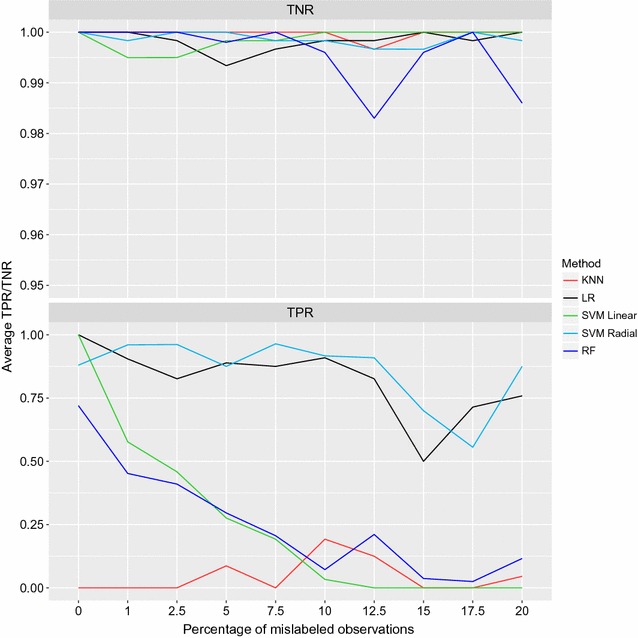
TPR (sensitivity) and TNR (specificity) as a function of noise in the data. Proportion of true negatives—i.e. non-carriers of the mutation-and true positives—i.e. carriers of the mutation, below—correctly identified (TNR and TPR) by the five methods over 10 error proportions. Lasso* black*, SVML* green*, SVMR* light blue*, RF* blue*, KNN* red*

The TNR (specificity) and TPR (sensitivity) for the five classification methods over the 10 proportions of errors are shown in Fig. 3. All methods showed a power of detecting non-carriers of the mutation (TNR) above 98%, with very small variation with increasing amounts of errors. KNN always attained 100% TNR, except at 12.5% noise. SVML and SVMR had an opposite behaviour: the former showing some false positives when the noise proportion was below 10% and the latter when the proportion was above 5%. RF showed the largest variability of TNR (98.3–100%).

The TPR (sensitivity) for the five methods shows much larger proportions of errors in response to increasing noise in the data. SVML correctly identified all carriers of the mutation only when no errors were introduced in the training set. As the noise proportion increased, TPR approached 0 (i.e. no detection power). A similar trend was shown by RF, which started at* TPR* = 72% with no mislabelings, and plummeted to* TPR* = 2.5% when 17.5% mislabeled observations were introduced in the data. For SVMR, TPR ranged between a minimum of 88% and a maximum of 96% when the noise proportion was below 15%, dropping to 55% when the error rate was 17.5%. Lasso showed a very similar TPR pattern as SVMR, with false negatives in the range 0–17% up to 12.5% noise, thereafter jumping to 50% false negatives, and finally relapsing to about 25%. The TPR for KNN was constantly very low (0–19%), irrespective of the amount of noise in the data. Table 1 reports the reciprocals of ACC (total error rate: $TER=1-ACC$), TNR (false positive rate: $FPR=1-TNR$) and TPR (false negative rate: $FNR=1-TPR$).

**Fig. 4:**
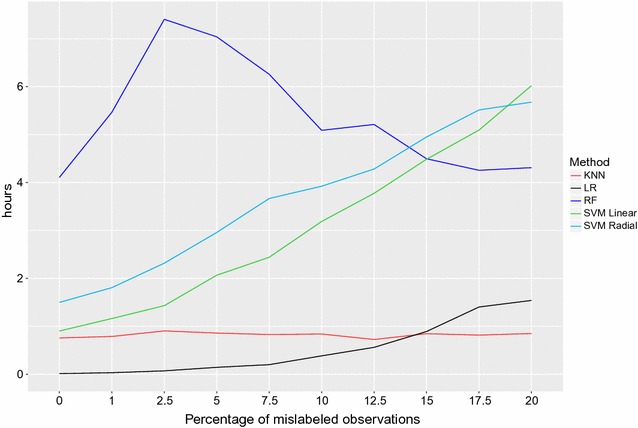
Computation time as a function of noise in the data. Computation time for the five classification methods and 10 proportions of mislabeled observations. Results from a 10-fold cross-validation procedure, repeated 10 times. Lasso* black*, SVML* green*, SVMR* light blue*, RF* blue*, KNN* red*

The total computation time for the five classification methods as a function of error percentage can be seen in Fig. 4. The elapsed time to run 10 times a 10-fold cross-validation scheme ranged from a minimum of 45 min in the scenario with no errors in the training set using KNN to a maximum of 7 h and 24 min using RF with 2.5% errors in the training set. The computation time remained more or less stable for KNN and Lasso over noise thresholds, while it increased approximately linearly with noise both for SVML and SVMR. RF required large computation times at all noise thresholds. Overall, RF was the most computationally demanding algorithm, followed by the two SVM implementations. SVML and SVR took longer than RF only with >15% noise in the data. Only with 20% noise in the data SVML took longer than SVMR to run.

## Discussion

In this paper, we presented how five classification methods responded to noise in the target variable. We selected two “global” linear methods (Lasso and SVML) and three “local” non-linear methods (KNN, RF and SVMR) in order to explore possible scenarios with state-of-the-art classification methods, each with specific properties.

The overall prediction accuracy in the base scenario (no noise) was close to $100\%$ with all five classification methods (with KNN providing the lower bound at $96\%$). However, the overall accuracy is known to be biased upwards when data are unbalanced [35], as is the ratio between carriers and non-carriers of the *TUBD1* mutation ($4\%$/$96\%$). In such cases, the proportion of errors in the two classes (carriers/non-carriers) gives a better representation of the relative performance of classifiers. In the analysed problem, the true positive and true negative ratios highlight the difficulty of correctly identifying carriers of the mutation (true positives), i.e. of predicting unobserved examples belonging to the minority class. All five methods identified non-carriers with virtually 100% accuracy (*TNR* = 100%), but they display different behaviour with respect to the prediction of carriers: KNN had a TPR very close to 0%, and never above 20%. With SVML, TPR was 100% with no noise in the data, and then rapidly decreased with increasing errors in the labels, eventually approaching* TPR* = 0 for noise >12.5%. RF followed a similar pattern, with a starting TPR of 72% that quickly decreased below 25% with minimum around 3%. SVMR and Lasso proved to be relatively more robust to noisy labels in the classification of mutation carriers: their TPR was larger than 80% up to 12.5% noise in the data, and only for larger proportions of errors in the data these two classifiers began to be unreliable. Standard classification algorithms have been shown to perform poorly with unbalanced data, and strategies to deal with unbalancedness have been proposed to improve the prediction accuracy [36, 37].

The worse relative performance of KNN, besides imbalance in the data, can be explained by the difficulty to handle large feature spaces: KNN is known to particularly suffer from the “curse of dimensionality” [38], especially when predictors are collinear, which can well be the case for SNP loci on the same chromosome, likely to be in moderate to high linkage disequilibrium (LD) with each other. The average pairwise LD between SNP loci on BTA19 was estimated as $r^2=0.126$ [8]. Support vector machines, focussing chiefly on pivotal training observations that define the classification margin (support vectors), are much less affected by high dimensional data. The selection of an appropriate kernel function is however important, since it defines the transformed feature space in which the training set instances will be classified. At base scenario (no noise), SVML outperformed SVMR and had, together with Lasso, a TPR of 100%. This indicates that the decision boundary in this problem is very likely linear, and a method like SVMR—which is known to potentially produce highly non-linear decision boundaries—is expected to perform relatively worse. When errors were introduced in the data, though, the ability of SVMR to accommodate non-linear relationships appeared to be helpful in maintaining relatively high predictive ability.

When introducing incremental percentages of errors in the labels (mislabeled carriers and non-carriers of the mutation), the dataset becomes increasingly noisy, and the task of correctly identifying true carriers and true non-carriers gets more challenging. The overall accuracy decreased as more errors were introduced, but on the whole seemed quite robust to mislabeled observations. This was however true for the accuracy of classifying observations belonging to the majority class (non-carriers), which is trivial with unbalanced data: the TNR remained above 99% irrespective of the amount of noise introduced in the data. On the other hand, the classification of carriers (minority class) gave a very different picture: with TPR suffering much more from noise in the data.

The use of a radial rather than a linear kernel in SVM seemed to make the classification more robust to errors in the labels. With increasing noise, the TPA, TNR and TPR curves became more wiggly, and higher accuracy in the testing rather than the training set was sometimes observed (results not shown: see [8]). When data get noisier, it is more difficult for predicting algorithms to classify observations correctly, as shown also by the increased computation time (Fig. 4); after a certain proportion of errors in the data, predictive models may break down and yield unreliable results (garbage in, garbage out: [39]). In the present dataset, this appeared to happen after 12.5% mislabeled observations in the training data.

**Table 1:** Total error rate (TER), false positive (FPR) and false negative (FNR) rates for the five classification models over the ten thresholds of random noise introduced in the data

Threshold	Variable	KNN	LR	RF	SVM linear	SVM radial
0.0000	TER	0.0401	0.0000	0.0112	0.0000	0.0048
0.0000	FPR	0.0000	0.0000	0.0000	0.0000	0.0000
0.0000	FNR	1.0000	0.0000	0.2800	0.0000	0.1200
1.0000	TER	0.0369	0.0032	0.0219	0.0225	0.0032
1.0000	FPR	0.0000	0.0000	0.0000	0.0050	0.0017
1.0000	FNR	1.0000	0.0952	0.5480	0.4231	0.0400
2.5000	TER	0.0417	0.0080	0.0236	0.0257	0.0016
2.5000	FPR	0.0000	0.0017	0.0000	0.0050	0.0000
2.5000	FNR	1.0000	0.1739	0.5900	0.5417	0.0385
5.0000	TER	0.0337	0.0096	0.0301	0.0353	0.0048
5.0000	FPR	0.0000	0.0066	0.0020	0.0017	0.0000
5.0000	FNR	0.9130	0.1111	0.7040	0.7241	0.1250
7.5000	TER	0.0338	0.0080	0.0318	0.0353	0.0032
7.5000	FPR	0.0000	0.0033	0.0000	0.0017	0.0017
7.5000	FNR	1.0000	0.1250	0.7940	0.8077	0.0357
10.0000	TER	0.0338	0.0048	0.0410	0.0465	0.0048
10.0000	FPR	0.0000	0.0017	0.0040	0.0000	0.0017
10.0000	FNR	0.8077	0.0909	0.9280	0.9667	0.0833
12.5000	TER	0.0482	0.0080	0.0479	0.0498	0.0064
12.5000	FPR	0.0034	0.0017	0.0170	0.0000	0.0033
12.5000	FNR	0.8750	0.1739	0.7890	1.0000	0.0909
15.0000	TER	0.0418	0.0209	0.0424	0.0417	0.0177
15.0000	FPR	0.0000	0.0000	0.0040	0.0000	0.0034
15.0000	FNR	1.0000	0.5000	0.9630	1.0000	0.3000
17.5000	TER	0.0482	0.0144	0.0390	0.0385	0.0193
17.5000	FPR	0.0000	0.0017	0.0000	0.0000	0.0000
17.5000	FNR	1.0000	0.2857	0.9750	1.0000	0.4444
20.0000	TER	0.0338	0.0112	0.0488	0.0465	0.0064
20.0000	FPR	0.0000	0.0000	0.0140	0.0000	0.0017
20.0000	FNR	0.9545	0.2414	0.8840	1.0000	0.1250

If computation time is also considered, Lasso provided the best combination in terms of classification accuracy and use of computer resources. SVMR showed comparable accuracy, but took much longer at base scenario and, especially, with noise in the data. RF was confirmed to be a demanding algorithm in terms of computing resources (see for instance Nazzicari et al. [40] for imputation of missing genotypes), unless computation strategies like parallelization are adopted; however, RF computation time seemed to be unaffected by noise in the data.

This paper focussed on the different behaviour of some standard machine/statistical learning methods for classification in response to mislabeled observations. When data are noisy, however, active strategies may be adopted to counteract—at least partially—the detrimental effect of noise on results from the statistical analysis: (a) data could be carefully cleaned before analysis [41]; (b) the loss functions by which the predictive equations are optimized can be modified to accommodate errors in the data e.g. by modelling explicitly or implicitly random and non-random errors [12, 42]; (c) locally adaptive approaches may be used to minimize the impact of errors in the data [43, 44].

## Conclusions

Machine learning methods have many applications and are gaining increasing popularity also in animal genetics. Data coming from animal recording are not free from errors or inconsistencies. The advent of precision livestock farming and automated data collection can on one hand alleviate the problem of manual or clerical errors, but may on the other hand introduce new sources of noise e.g. random spurious errors, bias in the machine, lack of double checking for errors. When such data are used for predictions, aspects related to the presence of noise have to be taken into account.

This work was the first to assess the impact of phenotyping errors on the accuracy of genomic predictions in animal genetics. The choice of the method used for predictions can influence results, being more or less susceptible to noise. With the present problem of classifying mutation carriers from SNP genotypes, SVM with radial kernel performed relatively well even when the proportion of errors in the data reached 12.5%. Lasso was the second best method, while SVML, RF and KNN were very sensitive to noise (KNN also to data unbalancedness). Taking into account both accuracy and computation time, Lasso provided the best combination among the options considered here (Additional file 1).

## Additional file

**Additional file 1.** R script used for classification with different thresholds of mislabeled observations.

## Abbreviations

SNP: single nucleotide polymorphism
KNN: K-nearest neighbors
Lasso: Lasso-penalised logistic regression
SVM: support vector machines
SVML: SVM with linear kernel
SVMR: SVM with radial basis function kernel
RF: random forest
ACC: total accuracy
TPR: true positive rate
TNR: true negative rate
